# Ten Simple Rules To Combine Teaching and Research

**DOI:** 10.1371/journal.pcbi.1000358

**Published:** 2009-04-24

**Authors:** Quentin Vicens, Philip E. Bourne

**Affiliations:** 1 University of Colorado, Boulder, Colorado, United States of America; 2 Skaggs School of Pharmacy and Pharmaceutical Science, University of California San Diego, La Jolla, California, United States of America

The late Lindley J. Stiles famously made himself an advocate for teaching during his professorship at the University of Colorado: “If a better world is your aim, all must agree: The best should teach” (http://thebestshouldteach.org/). In fact, dispensing high-quality teaching and professional education is the primary goal of any university [1]. Thus, for most faculty positions in academia, teaching is a significant requirement of the job. Yet, the higher education programs offered to Ph.D. students do not necessarily incorporate any form of teaching exposure. We offer 10 simple rules that should help you to get prepared for the challenge of teaching while keeping some composure.

## Rule 1: Strictly Budget Your Time for Teaching and for Doing Research

This rule may seem straightforward, but respecting it actually requires more discipline and skill than it first appears to. The key is to set aside time for both teaching and research from the beginning, with a well-marked separation (e.g., mornings will be devoted to course preparation, afternoons to experiments and manuscript writing). Firmly stick to this agenda, particularly if this is your first time teaching. Failure to do so would eventually affect the quality of your teaching or the progress of your research (or both). Over time, you will become more skilled at jumping from one commitment to the other, and therefore allowing the boundaries to fluctuate somewhat. Avoid underestimating the time necessary to fulfill teaching-related obligations (e.g., office hours, test preparation, grading, etc.) by consulting with your colleagues.

## Rule 2: Set Specific Teaching and Research Goals

In order not to have one occupation overpower the other one—which would transgress Rule #1—it is a good idea to decide on specific aims for each enterprise. Compile a list of reasonable but specific long-term goals (for the month or the semester) and short-term ones (for the week) for both your teaching (e.g., finish Chapter 3 by Nov. 1; this week propose a discussion to engage students to brainstorm about the risks of GMOs) and your research (e.g., finish experiments for this project and start writing before Easter; this week do the control for my primer binding assay). Make sure you achieve them. If you don't—this is likely to happen at first—ask yourself how legitimate your reason is. Then review and adjust the goals accordingly.

## Rule 3: “Don't Reinvent the Wheel”

We borrowed the title for this rule from excellent suggestions on *How To Prepare New Courses While Keeping Your Sanity* [2]. Most likely, you will not be the first one ever to teach a particular topic. So get in touch with the colleagues in your department who have taught the class you are going to teach, or who teach similar topics. You can also use your network and contact former colleagues or friends at other institutions. They will usually be happy to share their course material, and along the way you might also glean precious tips from their teaching experience (e.g., a list of do's and don'ts on how to approach a notoriously difficult topic). You will also learn a lot from sitting in one of their classes and watching how they handle their topic and their students. Here are more examples of precious time-savers:

1. Choose a textbook that is accompanied by rich online resources such as annotated figures, pre-made PowerPoint slides, animations, and videos. Students will thank you for showing movies, for example, as they often are a better option to break down complex mechanisms or sequences of events into distinct steps.
2. Administer a Web site for your course. Many universities and some textbooks now offer you the possibility of hosting a Web site with course-related materials, including automatically graded assessments. See, for example, the CULearn suite used at the University of Colorado (http://www.colorado.edu/its/culearn/), or more general automatic grading tools presented at http://ctl.stanford.edu/Tomprof/postings/227.html.
3. Gather a solid team of motivated teaching or learning assistants, who will both serve as an intermediary between you and your students and help you grade. In short, don't be afraid to ask for help!

## Rule 4: Don't Try To Explain Everything

Class time should be spent guiding students to create their own explanation of the material and to develop cognitive abilities that will help them become critical thinkers. In other words, you don't want to present all aspects related to a certain topic or to lay out all the explanations for them. Thus, an effective way to teach is to get students to learn by transformative learning: beyond memorizing and comprehending basic concepts, they will learn to reflect on what they learn and how they learn it (see, for example, http://en.wikipedia.org/wiki/Transformative_learning and references within). Such teaching practices require that a significant part of the learning process happens outside the classroom, through reading assignments, homework, writing essays, etc. So make sure you budget time to organize these, as specified in Rule #2. Remember that in the end this will be a win–win situation: you will save time by not having to fit everything into your class time, and students will learn how to find answers through their own thinking.

## Rule 5: “Be Shameless in Bringing Your Research Interests into Your Teaching”

This is yet another great time-saver, and this rule title is actually from *Confessions about Stress and Time: Thoughts for Faculty* (available at http://www.colorado.edu/ftep/publications/confessions.html). Students want to know how what you teach relates to the world around them. They also like to know what is happening in science right now, so this is where you can feed in some of your research interests (for some examples of how researchers around the world have been bringing their research into the classroom, refer to the special section of the July 6, 2007, issue of the magazine *Science* entitled *The World of Undergraduate Education* [3]. Students will welcome such connections, especially in an introductory course or in a course for non-majors. Additionally, they will feel the passion that makes you love being a scientist. On your end, you might find that preparing course materials will be easier (because you are already a master of that topic), and you might learn to be more comfortable at presenting your research in layman's terms.

## Rule 6: Get the Most in Career Advancement from Bringing Your Research into Your Teaching

As a sort of followup to Rule #5, presenting your research in class could bring you a solid return on your investment. For example, teaching gives you exposure; talking about your research may help you recruit motivated students in your lab, which will help you advance your research, possibly by taking it in original directions. In parallel, you could also use your research to design a novel course and possibly evaluate student learning in a fashion that would make for a publication in a science education journal. Another option would be to write or edit a book, or to contribute a chapter in someone else's book that you would eventually give as a reading assignment in your class. Conversely, there is wisdom in crowds. Consider having students review aspects of your research that fit the course and get feedback. You will be surprised at what useful information can come from students critiquing a new manuscript or proposing new experiments.

## Rule 7: Compromise, Compromise, Compromise

A significant part of the compromise once you accept a joint research/teaching commitment is to realize that your list of “things that in principle you would like to do but won't have time to do” will get longer. Maybe you would like to personally respond to all the students who e-mail you about any problem they may have, but, realistically, such things can't happen. Instead, a solution would be to send some general feedback in answer to the common queries and to write occasional brief personal responses. As you get more skilled at combining research and teaching, you will be able to progressively bring back activities such as scanning the most recent scientific literature and attending seminars and lectures more often. But remember to accept that no matter how skilled you are at budgeting your time for teaching and research, you will still face the conflicting demands of both, and you will have to keep compromising. In the end, compromising will sometimes imply learning to say no when pondering about taking on a novel and exciting assignment that would unequivocally conflict with your current research/teaching agenda.

## Rule 8: Balance Administrative Duties with Your Teaching and Research Workload

Your responsibility as a teacher and as a researcher is to be as productive as you can be in these two areas, at the same time. This is what your colleagues and the faculty board will expect from you when evaluating you for tenure, for example. Doing service within your community (for example by sitting on committee meetings, or by being part of a local scientific club) counts as well, but not as much. In consequence, turning down yet another offer to organize a series of seminars, or to edit the newsletter of your department, is legitimate if it cuts into your productivity. Similarly, keep your ability to career advance in mind when considering taking on another teaching assignment.

## Rule 9: Start Teaching Early in Your Career

This will be the best way to get exposed to some of the difficulties mentioned in the other Rules sooner rather than later. You can see this as an opportunity to learn how to add on various responsibilities in a gradual rather than an immediate manner (e.g., when “jumping” from a post-doc to a faculty position at a university). Many options are available to teach at the graduate level (e.g., by becoming a teaching/learning assistant), as well as at the post-graduate level (e.g., by teaching part-time on campus or at a local school while doing your post-doc). You may need to be proactive about looking for such opportunities, but an increasing number of universities and institutions are developing programs that formally offer teaching experience to graduate students and post-docs [4],[5].

## Rule 10: Budget Time for Yourself, Too

A lot of stress can build up from a constant shuttle between teaching demands and research occupations. In order to be able to evacuate some of that tension, it is a good idea to hide some time for yourself that you will spend with your family, or to do your hobby, to exercise, to travel, etc. An unfulfilling personal life is incompatible with successful teaching and research careers. Consequently, don't forget to spend some energy learning how to balance both areas.

Finally, keep in mind that your experience can make for a valuable contribution to the scientific community, for example, in the form of a report on your efforts in science education, or by posting comments to this Editorial!
